# Enhanced Biosynthesis of Withanolides by Elicitation and Precursor Feeding in Cell Suspension Culture of *Withania somnifera* (L.) Dunal in Shake-Flask Culture and Bioreactor

**DOI:** 10.1371/journal.pone.0104005

**Published:** 2014-08-04

**Authors:** Ganeshan Sivanandhan, Natesan Selvaraj, Andy Ganapathi, Markandan Manickavasagam

**Affiliations:** 1 Department of Biotechnology and Genetic Engineering, School of Life Sciences, Bharathidasan University, Tiruchirappalli, Tamil Nadu, India; 2 Govt. Arts College, Kulithalai, Tamil Nadu, India; Indiana University, United States of America

## Abstract

The present study investigated the biosynthesis of major and minor withanolides of *Withania somnifera* in cell suspension culture using shake-flask culture and bioreactor by exploiting elicitation and precursor feeding strategies. Elicitors like cadmium chloride, aluminium chloride and chitosan, precursors such as cholesterol, mevalonic acid and squalene were examined. Maximum total withanolides detected [withanolide A (7606.75 mg), withanolide B (4826.05 mg), withaferin A (3732.81 mg), withanone (6538.65 mg), 12 deoxy withanstramonolide (3176.63 mg), withanoside IV (2623.21 mg) and withanoside V (2861.18 mg)] were achieved in the combined treatment of chitosan (100 mg/l) and squalene (6 mM) along with 1 mg/l picloram, 0.5 mg/l KN, 200 mg/l L-glutamine and 5% sucrose in culture at 4 h and 48 h exposure times respectively on 28^th^ day of culture in bioreactor. We obtained higher concentrations of total withanolides in shake-flask culture (2.13-fold) as well as bioreactor (1.66-fold) when compared to control treatments. This optimized protocol can be utilized for commercial level production of withanolides from suspension culture using industrial bioreactors in a short culture period.

## Introduction

Plants produce a wide range of secondary metabolites, which are used for a number of applications, such as pharmaceuticals, bio-pesticides, flavors, fragrances, colors and food additives. At present, these products have been obtained from plants growing in the wild or cultivated sources. The utilization of huge quantities of whole plants raised a major question that it can minimize local plant populations and erode their genetic diversity. Moreover, the plants growing in the wild suffer from various climatic and environmental fluxes which bring changes in their chemical profile. In recent years, there has been a renewed interest in the use of medicinal plants and medicinal plant products as an alternative to synthetically produced pharmaceuticals for the prevention and treatment of ailments and diseases, which resulted in the growth of industries producing nutraceuticals and pharmaceuticals every year [1]. Plant cell/organ cultures have been perceived as promising choice to traditional plant extraction for obtaining valuable chemicals throughout the year. Particularly, cell suspension culture offers a condensed biosynthetic cycle to study the growth and production kinetics within a short cultivation time (about 2–4 weeks) with an added advantage of tunability that can help to implement optimal conditions for the production of a number of high value medicinal compounds in good quantities [2], [3].

The medicinal importance of *Withania somnifera* (*Solanaceae*) is mainly because of the presence of steroidal lactones namely “withanolides” [4], [5]. The pharmaceutically important compounds are withanolide A, withanolide B, withaferin A, and withanone (major constituents) and 12-deoxy withastramonolide, withanoside IV and withanoside V (minor constituents). Each withanolide is having a wide array of therapeutic values. For instance, withanolide A is considered as a good candidate for neurodegenerative diseases and potentiating humoral and cell mediated Th1 immunity [6], [7], [8]. Withaferin A induces apoptosis through ROS generation mediating modulation of both intrinsic and extrinsic apoptosis signalling cascades together with abrogation of NF-kB functions [9], antiplatelet activities [10], anti-inflammatory, cardiovascular protection, anti-cancer, anti-oxidant activities [11] besides being strongly antiangiogenic and anti-metastatic [12]. Withanolide B, withaferin A and withanone have remarkable activities in physiological and metabolic restoration, anti-arthritic, anti-aging, anti-cancer, cognitive function improvement in geriatric states and recovery from neurodegenerative disorders [13].

The requirement of dried plant material for withanolides production in India has been estimated to about 9, 127 tonnes as against the annual production of about 5, 905 tonnes [14], [15]. A major bottleneck in the biosynthesis of withanolides depends on plant's tissue type and growth conditions in natural habitats as wide commercial products are entirely derived from field-grown plants [16]. This ultimately leads to difficulties in the compositional standardization of *Withania* formulations and its commercial exploitation [16]. The traditional cultivation of *W. somnifera* with respect to withanolides drug preparation has been limited by a range of issues such as biotic and abiotic environmental factors, unpredictability of bioactive components synthesis and lack of purity and standardized plant's raw material for phytochemical analysis. In addition, these methods are time consuming, laborious and they are not able to meet the current ashwagandha global market requirement [15]. At the international level, there has been an ever-increasing demand for *W. somnifera* in larger quantities [15].

Plant cell, tissue and organ culture systems offer alternative platform for the therapeutically valuable secondary metabolite production. Zhang et al. [17] put forth that plant cell culture has been declared as a feasible tool in producing numerous plant-derived metabolites in higher quantities. *In vitro* plant cell suspension culture facilitates large-scale production of fine chemicals in industrial bioreactors and for the study of cellular and molecular processes as it offers the advantage of a simplified model system for studying physiological effect of salt at the cellular level under a controlled environment [18] and the effect of heavy metal stress on growth, enzymes activities and altered biochemical parameters in cultured cells [19]. Cell suspension cultures contain a relatively homogeneous cell population, allowing rapid and uniform access to nutrition, precursors, growth hormones and signal compounds in the cells [20].

However, commercial utilization of plant cell cultures has been met with limited success and restricted to a few secondary metabolites [21], [22]. Low or no product yield, biosynthetic instability and difficulties to scale-up are some of the major reasons for an unforeseen commercial recovery of secondary metabolites in cell cultures [23]. To solve this problem, screening and selection of high-producing cell line, medium optimization, elicitation, precursor feeding, *in situ* product removal, and immobilization [21], [24], [25], [26] strategies can be manipulated for the feasible production of secondary metabolites in plant cell/organ cultures. Among these, precursor feeding is one of the novel techniques to over express genes involved in biosynthesis pathway. In the case of *W. somnifera*, withanolides are biosynthesized via two different pathways namely mevalonate pathway and non-mevalonate pathway. In the withanolides metabolic pathway, cholesterol, mevalonic acid and squalene are the intermediate precursors. Feeding the precursors in a metabolic pathway has shown to increase the amount of compounds produced by suspension culture [27].

Elicitation is also one of the effective methods to improve secondary metabolite production in cell and organ culture. We have investigated a variety of biotic and abiotic elicitors for the enhancement of major and minor withanolides production in multiple shoots, adventitious roots and hairy root cultures of *W. somnifera*, and a number of effective biotic and abiotic elicitors were established [5], [15], [28], [29]. In addition, we obtained higher biomass accumulation and withanolides profile production in cell suspension culture when compared to field-grown plants of *W. somnifera* by the influence of auxin, cytokinins, agitation speed, nitrogen sources, carbon sources and seaweed extracts [28] in contrast to a few reports on low production of withanolide A and withaferin A in cell suspension culture [30]–[34]. In the present study, we assessed the combined effects of elicitors and precursors on withanolides production in cell suspension culture utilizing shake-flask culture system as well as bioreactor culture system in *W. somnifera* for the first time.

## Materials and Methods

### Plant material and establishment of callus culture

Roots from 4-week-old *in vitro* seedlings were used as explant source for initiation of callus. Callus culture was established according to our previous report [28]. The roots were excised into small segments (10–15 mm length) and aseptically transferred to MS [35] medium augmented with 2 mg/l picloram. All the cultures were maintained on same composition of the medium and subcultured every 3 weeks for 6 weeks. The pH of the medium was adjusted to 5.8 by 0.1 N NaOH/HCl prior to addition of 0.8% agar. The medium was autoclaved at 121°C at 15 lbs for 20 min. The cultures were maintained under total darkness at 25±2°C for 6 weeks.

### Kinetics of biomass accumulation and withanolides production in shake-flask culture

The cell suspension cultures were initiated by inoculating ∼500 mg fresh mass of friable callus actively dividing cells at the upper surface in 150 ml Erlenmeyer flask containing 30 ml of MS liquid medium supplemented with 1 mg/l picloram, 0.5 mg/l KN, 200 mg/l L-glutamine and 5% sucrose and kept on a gyratory shaker at 120 rpm under total darkness [28]. To estimate growth and production kinetics, the cultures were harvested in duplicate at a time period of 7, 14, 21, 28, 35 and 42 days and analyzed for FW, DW and withanolides production.

### Impact of elicitors on biomass accumulation and withanolides production in shake-flask culture

Stock solutions of aluminium chloride, and cadmium chloride were prepared separately by dissolving them in sterile water and adjusting their pH 5.7. Chitosan was dissolved as per our earlier report [5]. All the elicitors were aseptically added to the culture medium at the following concentrations, aluminium chloride, cadmium chloride and chitosan – 5, 10, 15, 20, 25 mg/l and 50, 100, 150, 200, 250 mg/l respectively at exposure times of 0, 2, 4, 6, 8 h. The elicitors were added on the 21^st^ day of culture. All the cultures were harvested on the 28th day (since this day showed maximum production kinetics on biomass accumulation and withanolides production) and analyzed for biomass accumulation and withanolides production. Control cultures were maintained for each experiment.

### Impact of precursor feeding on biomass accumulation and withanolides production in shake-flask culture

Three different precursors such as mevalonic acid, cholesterol and squalene were used at different concentrations (0–250 µM and 0–10 mM) respectively with various exposure times (0, 12, 24, 36, 48, 60, 72 h) in culture. Stock solutions of mevalonic acid, cholesterol and squalene were prepared in acetone and 99% ethanol respectively. The precursors were added on the 21st day of culture. All the cultures were harvested on the 28th day (after 7 days; since this day showed maximum production kinetics on biomass accumulation and withanolides production) and analyzed for biomass accumulation and withanolides production. Appropriate control was maintained for each experiment.

### Productivity improvement by bioprocess in bioreactor

The data obtained after optimization of precursor feeding and elicitation at the optimal exposure time were integrated in order to manipulate a bioprocess system for the improvement of withanolides productivity in 7-l bioreactor by inoculating ∼83 g fresh mass of friable callus. The MS liquid medium (5-l working volume) consisted of optimal concentrations of picloram (1 mg/l), KN (0.5 mg/l), L-glutamine (200 mg/l) and 5% sucrose at 25±2°C under total darkness. Precursor (squalene 6 mM) and elicitor (chitosan 100 mg/l) were added individually on 21^st^ day with 48 h and 4 h exposure times (optimum exposure times) respectively. The cultures were harvested on the 28th day (after 7 days) and analysed for biomass accumulation and all major and minor withanolides (withanolide A, withanolide B, withaferin A, withanone, 12-deoxy withastramonolide, withanoside IV and withanoside V) in cell suspension culture.

### Determination of biomass accumulation

Biomass accumulation was estimated as per our earlier report [28]. The cell suspensions were separated from the medium by filtering them and fresh weight (FW) was measured after rinsing with sterile water and blotting away surface water. The dry weight (DW) was recorded after the cell suspensions were dried constantly at 60°C in oven for 2 days.

### Withanolides extraction and HPLC analysis

Extraction and HPLC analysis were performed as described by Sivanandhan et al. [28]. Standard samples of withanolides A and B were obtained from Chromadex Inc. (Laguna Hills, CA, USA) and withaferin A, withanone, 12 deoxy withanstramonolide, withanosides IV and V were obtained from Natural Remedies (Bangalore, Karnataka, India).

### Statistical analysis

A completely randomized design was used for all treatments. All the experiments were repeated thrice with three replicates for each treatment. Data were statistically analyzed using analysis of variance (ANOVA). Data were presented as mean±standard error (SE). Values wih the same letter are not significantly different. The mean separations were carried out using Duncan's multiple range test and significance was determined at 5% level (SPSS 11.5).

## Results

### Kinetics of biomass accumulation and withanolides production in shake-flask culture and bioreactor

**Figure 1:**
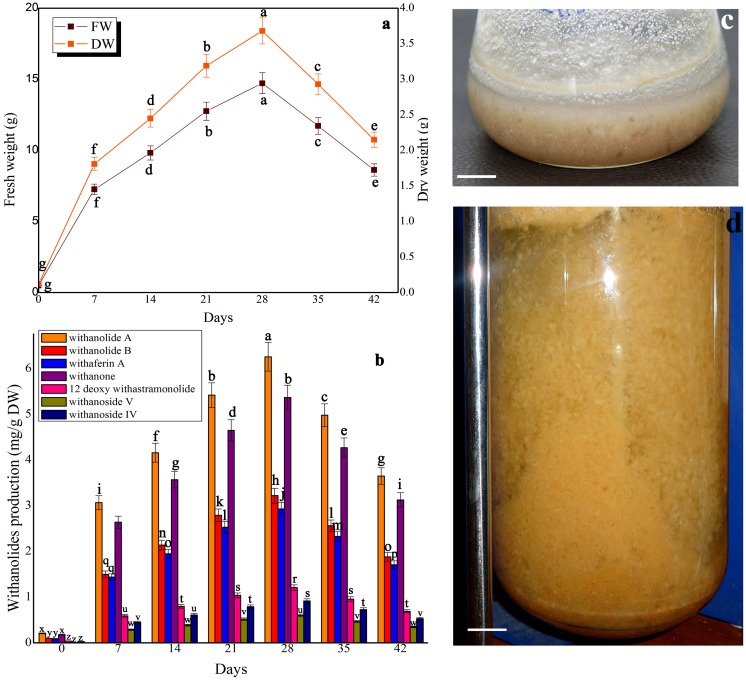
Growth kinetics and withanolides production in cell suspension culture of *W. somnifera*. a. Dynamic profiles of biomass accumulation, b. Dynamic profiles of withanolides production, c. Synchronized cell suspension culture in 150-flask culture, d. Lab scale cell suspension culture in 7-l bioreactor. The cell suspension cultures were established by inoculating ∼500 mg fresh mass of friable callus in 150 ml Erlenmeyer flask containing 30 ml of MS liquid medium supplemented with 1 mg/l picloram, 0.5 mg/l KN, 200 mg/l L-glutamine and 5% sucrose and kept on gyratory shaker at 120 rpm under total darkness. Bioreactor culture was established with 83 g FW of friable callus in 5-l MS liquid medium with same hormonal combinations. Values represent mean ±standard error of three replicates; each experiment was repeated thrice. All bars = 1 cm.

The biomass accumulation (FW and DW) and withanolides (withanolide A, withanolide B, withaferin A, withanone, 12 deoxy withanstramonolide, withanosides IV and V) production in MS medium containing picloram, KN, L-glutamine and sucrose are shown in figure 1a and 1b. The growth of cells in suspension culture exhibited two phases. Upon inoculum feeding in the suspension medium, the cell mass continued to increase particularly between days 7–28 which could be an equivalent of “exponential growth phase” followed by a death phase (days 29–42) [Figure 1a, c, d]. The biomass reached a maximum with a fresh weight of 14.72 g and a dry weight of 3.68 g on 28^th^ day (Figure 1a). All withanolides were produced during the exponential growth phase (7–28 days) followed by a decline during death phase (29–42 days). However, such a decline was still higher when compared to the levels of withanolides produced at 0 day of culture. On 28^th^ day of culture, maximum levels of withanolides were detected (withanolide A (6.26 mg/g DW), withanolide B (3.22 mg/g DW), withaferin A (2.93 mg/g DW), withanone (5.37 mg/g DW), 12-deoxy withanstramonolide (1.21 mg/g DW), withanosides IV (0.92 mg/g DW) and V (0.60 mg/g DW) [Figure 1b] and the withanolides detected as against the dry weight were withanolide A (23.03 mg), withanolide B (11.84 mg), withaferin A (10.78 mg), withanone (19.76 mg), 12-deoxy withanstramonolide (4.45 mg), withanosides IV (2.20 mg) and V (3.38 mg) [Table 1; Figure S1]. In bioreactor culture, a maximum biomass accumulation (1889.43 g FW and 472.35 g DW) was obtained on 28^th^ day of culture and the withanolides detected as against the dry weight were withanolide A (5800.45 mg), withanolide B (2961.63 mg), withaferin A (2494.00 mg), withanone (3991.35 mg), 12-deoxy withanstramonolide (1643.77 mg), withanosides IV (878.57 mg) and V (1058.06 mg) [Table 1; Figure S1].

**Table 1:** Productivity of withanolides in shake-flask culture and bioreactor by elicitor and precursor treatments in cell suspension of *W. somnifera*.

Treatment (Shake-flask culture)	Dry weight (g)	Total withanolide A detected (mg)	Total withanolide B detected (mg)	Total withaferin A detected (mg)	Total withanone detected (mg)	Total 12-deoxy withanstramonolide detected (mg)	Total withanoside V detected (mg)	Total withanoside IV detected (mg)	Total withanolides detected (mg)
Control	3.68±0.20e	23.03	11.84	10.78	19.76	4.45	3.38	2.20	75.44
Chitosan (100 mg/l)	2.63±0.21f	38.81 (1.69)	24.56 (2.07)	17.56 (1.62)	32.27 (1.63)	12.15 (2.73)	9.52 (2.81)	6.52 (2.96)	141.39 (1.87)
Squalene (6 mM)	2.94±0.27f	25.37 (1.1)	11.08 (−0.93)	3.98 (−0.36)	20.08 (1.01)	7.11 (1.59)	3.26 (−0.96)	2.88 (1.30)	73.76 (−0.97)
Squalene+Chitosan (6 mM+100 mg/l)	2.15±0.29f	37.88 (1.62)	26.53 (2.24)	20.94 (1.94)	33.71 (1.70)	15.67 (3.52)	13.50 (3.99)	12.59 (5.72)	160.82 (2.13)
Treatment (Bioreactor culture)									
Control	472.35±0.23a	5800.45	2961.63	2494.00	3991.35	1643.77	1058.06	878.57	18827.83
Chitosan (100 mg/l)	337.39±0.21c	7172.91 (1.23)	3195.08 (1.07)	3967.70 (1.59)	5678.27 (1.42)	2314.49 (1.40)	1902.87 (1.79)	1504.75 (1.71)	25736.07 (1.36)
Squalene (6 mM)	461.53±0.26b	7273.71 (1.25)	3179.94 (1.07)	3392.24 (1.36)	4467.61 (1.11)	2021.50 (1.22)	1513.81 (1.43)	1001.52 (1.13)	22850.33(1.21)
Squalene+Chitosan (6 mM+100 mg/l)	276.71±0.27d	7606.75 (1.31)	4823.05 (1.62)	3732.81 (1.49)	6538.65 (1.63)	3176.63 (1.93)	2861.18 (2.70)	2623.21 (2.98)	31362.28 (1.66)

For Shake-flask culture.

Initial inoculum – ∼500 mg cell mass for 30 ml MS medium.

For Bioreactor culture.

Initial inoculum – ∼83 g FW cell mass for 5 l MS medium.

Data were recorded on 28^th^ day of culture for both the experiments.

Values represent the mean ± standard error of three experiments. Mean values followed by the same letters within a column are not significantly different according to Duncan's multiple range test at 5% level.

### Impact of elicitors on biomass accumulation and withanolides production in shake-flask culture and bioreactor

**Figure 2:**
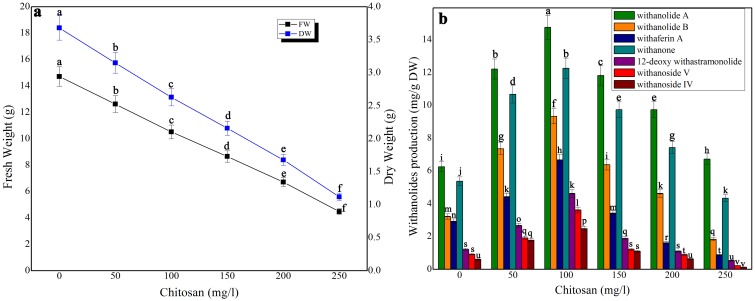
The effect of different concentrations of chitosan on growth characteristics (a) and withanolides production (b) in cell suspension culture of *W. somnifera* in shake-flask culture at 4 h exposure time. Five hundred milligram of fresh mass of friable callus was cultured in 30/l picloram, 0.5 mg/l KN, 200 mg/l L-glutamine and 5% sucrose and kept on gyratory shaker at 120 rpm under total darkness. The cultures were harvested on 28^th^ day. Data represents mean±standard error of three replicates; each experiment was repeated thrice.

**Figure 3:**
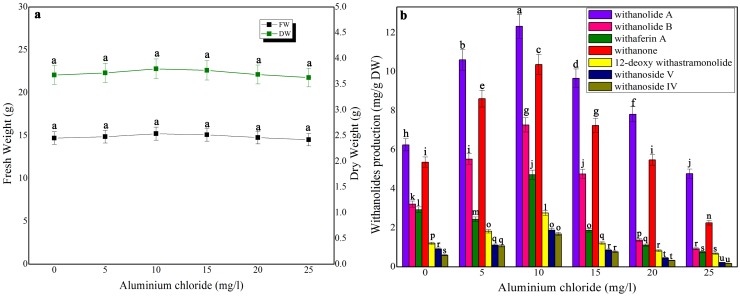
The effect of different concentrations of aluminium chloride on growth characteristics (a) and withanolides production (b) in cell suspension culture of *W. somnifera* in shake-flask culture at 4 h exposure time. Five hundred milligram of fresh mass of friable callus was cultured in 30/l picloram, 0.5 mg/l KN, 200 mg/l L-glutamine and 5% sucrose and kept on gyratory shaker at 120 rpm under total darkness. The cultures were harvested on 28^th^ day. Data represents mean±standard error of three replicates; each experiment was repeated thrice.

**Figure 4:**
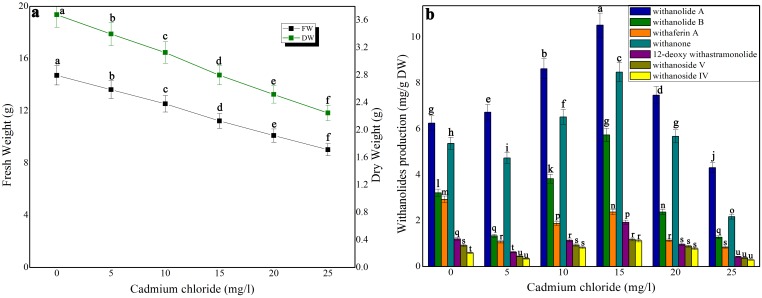
The effect of different concentrations of cadmium chloride on growth characteristics (a) and withanolides production (b) in cell suspension culture of *W. somnifera* in shake-flask culture at 4 h exposure time. Five hundred milligram of fresh mass of friable callus was cultured in 30/l picloram, 0.5 mg/l KN, 200 mg/l L-glutamine and 5% sucrose and kept on gyratory shaker at 120 rpm under total darkness. The cultures were harvested on 28^th^ day. Data represents mean±standard error of three replicates; each experiment was repeated thrice.

Three elicitors, namely, chitosan, cadmium chloride and aluminium chloride were tested for biomass accumulation and withanolides production in suspension culture at various times (Figures 2, 3, 4). Biomass accumulation of chitosan and cadmium chloride elicited (4 h exposure time) cell suspension cultures was gradually declined whereas in aluminium chloride treated cell suspension culture it remained stable. There was only a 12% growth decline in 50 mg/l chitosan-treated cell suspension culture. On the otherhand, at its higher concentrations (above 100 mg/l), biomass was significantly affected (Figure 2a). At 100 mg/l chitosan elicitation, maximum levels of withanolides [withanolide A (14.76 mg/g DW), withanolides B (9.34 mg/g DW), withaferin A (6.68 mg/g DW), withanone (12.27 mg/g DW), 12 deoxy withanstramonolide (4.62 mg/g DW), withanosides IV (2.48 mg/g DW) and V (3.62 mg/g DW)] were produced from the cells harvested on 28^th^ day of culture (Figure 2b) with the concentrations of total withanolides 141.39 mg. In bioreactor culture, the levels of total withanolides detected against dry weight were increased i.e 25736.07 mg (Table 1; Figure S1). Although aluminium chloride at 10 mg/l elicitation enhanced withanolides production without causing any significant changes in biomass accumulation (Figure 3a), its production on per gram dry weight basis was next only to chitosan's (100 mg/l) withanolides production (Figure 3b). Cadmium chloride at 15 mg/l at 4 h exposure time redued biomass accumulation and increased withanolides production which was next only to withanolides production in chitosan and aluminium chloride treated cell suspensions (Figure 4 a and b).

### Impact of precursor feeding on biomass accumulation and withanolides production in shake-flask culture and bioreactor

**Figure 5:**
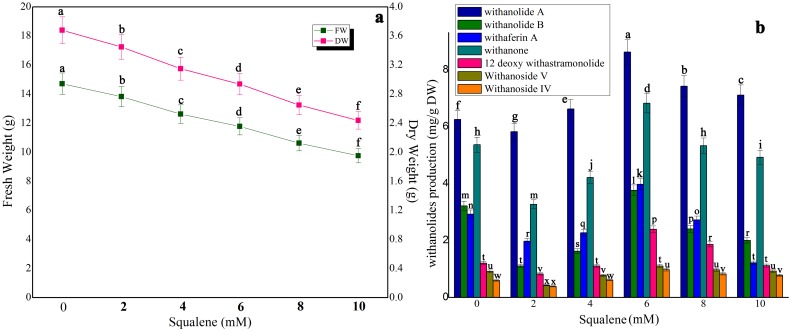
The effect of different concentrations of squalene on biomass accumulation (a) and withanolides production (b) in cell suspension culture of *W. somnifera* in shake-flask culture at 48 h exposure time. Five hundred milligram of fresh mass of friable callus was cultured in 30/l picloram, 0.5 mg/l KN, 200 mg/l L-glutamine and 5% sucrose and kept on gyratory shaker at 120 rpm under total darkness. The cultures were harvested on 28^th^ day. Data represents mean±standard error of three replicates; each experiment was repeated thrice.

**Figure 6:**
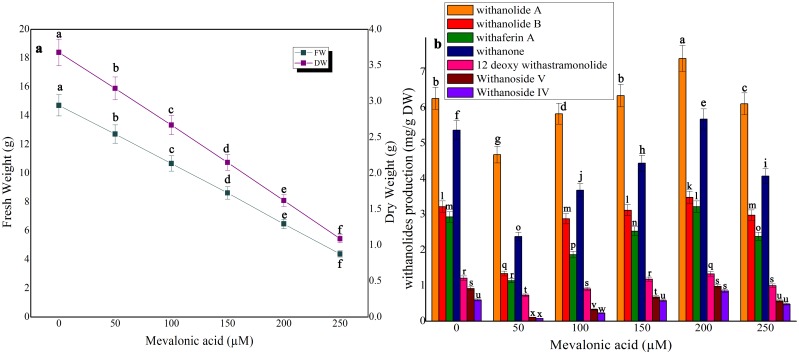
The effect of different concentrations of mevalonic acid on biomass accumulation (a) and withanolides production (b) in cell suspension culture of *W. somnifera* in shake-flask culture at 48 h exposure time. Five hundred milligram of fresh mass of friable callus was cultured in 30/l picloram, 0.5 mg/l KN, 200 mg/l L-glutamine and 5% sucrose and kept on gyratory shaker at 120 rpm under total darkness. The cultures were harvested on 28^th^ day. Data represents mean±standard error of three replicates; each experiment was repeated thrice.

**Figure 7:**
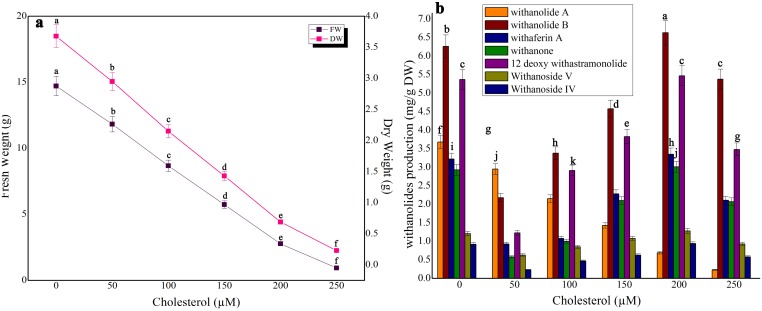
The effect of different concentrations of cholesterol on biomass accumulation (a) and withanolides production (b) in cell suspension culture of *W. somnifera* in shake-flask culture at 48 h exposure time. Five hundred milligram of fresh mass of friable callus was cultured in 30/l picloram, 0.5 mg/l KN, 200 mg/l L-glutamine and 5% sucrose and kept on gyratory shaker at 120 rpm under total darkness. The cultures were harvested on 28^th^ day. Data represents mean±standard error of three replicates; each experiment was repeated thrice.

Three precursors, namely, squalene, mevalonic acid and cholesterol were tested for biomass as well as withanolides production (Figures 5, 6, 7). The control treatment in shake-flask culture exhibited 14.72 g FW and 3.68 g DW whereas in bioreactor culture 1889.43 g FW and 472.35 g DW were obtained (Figure 5a and Table 1). In squalene-fed (48 h exposure time) shake-flask culture, the biomass was considerably reduced at all tested concentrations (Figure 5a). Squalene at 6 mM in shake-flask culture yielded the highest synthesis of withanolide A (8.63 mg/g DW; 1.37-fold), withanolide B (3.77 mg/g DW; 1.17-fold), withaferin A (3.98 mg/g DW; 1.35-fold), withanone (6.83 mg/g DW; 1.27-fold), 12 deoxy withanstramonolide (2.42 mg/g DW; 2.0-fold), withanosides IV (0.98 mg/g DW; 1.63-fold) and V (1.11 mg/g DW; 1.2-fold) when compared to control (Figure 5b). At the same concentration of squalene, the bioreactor culture yielded a concentration of withanolide A (7273.71 mg), withanolide B (3179.94 mg), withaferin A (3392.24 mg), withanone (4467.61 mg), 12 deoxy withanstramonolide (2021.50 mg), withanosides IV (1001.52 mg) and V (1513.81 mg) with total withanolides production of 22850.33 mg (Table 1; Figure S1). In the case of mevalonic acid, biomass accumulation was reduced at all concentrations (Figure 6a) and enhanced withanolides production at 200 µM but next only to squalene on mg/g DW basis [50–250 µM] (Figure 6). Cholesterol elicitation at different concentrations reduced biomass accumulation as well as withanolides production except at 200 µM where there was a moderate enhancement in shake-flask culture (Figure 7).

### Productivity improvement by bioprocess in bioreactor

Precursor, squalene (6 mM) and elicitor, chitosan (100 mg/l) concentrations were optimized for the production of all major and minor withanolides in bioreactor. The biomass and withanolides productivity in bioreactor were significantly higher when compared to shake-flask culture (Table 1). In addition, when aluminium chloride was combined with squalene, the biomass was affected and withanolides production was not better than the combined treatment of squalene and chitosan in shake-flask culture (Data not shown). Hence, the combined treatment of squalene and chitosan was tried in bioreactor setting. In shake-flask culture, the combination of squalene and chitosan resulted in 2.13-fold increase as against 1.66-fold increase in the total withanolides detected (Table 1). The dry weight of bioreactor culture on MS medium containing optimal concentrations of picloram and L-glutamine with the combined treatments of elicitor and precursor was 276.71 g in bioreactor (Table 1). The maximum withanolides detected against dry weight in bioreactor: withanolide A (7606.75 mg), withanolide B (4823.05 mg), withaferin A (3732.81 mg), withanone (6538.65 mg), 12 deoxy withanstramonolide (3176.63 mg), withanosides IV (2623.21 mg) and V (2861.18 mg) which were 1 to 3-folds higher when compared control culture in bioreactor (Table 1).

## Discussion

Ouyang et al. [36] stated that secondary metabolites were synthesized profusely after the cell growth entered into exponential phase. Hence, prior to adding precursors and elicitors into the culture medium, optimal culture period for maximum biomass yield and secondary metabolites production was tested. In control treatment, maximum biomass accumulation (14.72 g FW and 3.68 g DW) was achieved on 28^th^ day of culture. Liu et al. [37] explained that during lag phase, the plant cells needed to adjust to the new environment and during the exponential phase the plant cells promoted to accumulate maximum biomass accumulation and secondary metabolites production. In the present study, highest withanolides contents [withanolide A (6.26 mg/g DW), withanolide B (3.22 mg/g DW), withaferin A (2.93 mg/g DW), withanone (5.37 mg/g DW), 12-deoxy withanstramonolide (1.21 mg/g DW), withanosides IV (0.92 mg/g DW) and V (0.60 mg/g DW)] was recorded in the control at 28^th^ day of culture which exhibited exponential phase (7–28 days) in cell suspension culture of *W. somnifera*. The secondary metabolites productivity was entirely dependent up on the exponential phase of cell lines, nutrient medium supplemented with PGRs, nitrogen and carbon sources [2], [28]. Similar results were documented in *Cistanche salsa* [37] and *Artemisia annua* [38] cell suspension cultures.

In the present examination on elicitation, the influences of aluminium chloride, chitosan, cadmium chloride on withanolides biosynthesis in *W. somnifera* cell suspension cultures were examined. The elicitors were added at different concentrations with varied exposure times in the culture medium on 21 day-old cell suspension cultures. Among the three elicitors tested, chitosan enhanced the contents of total withanolides 1.87-fold higher in shake-flask culture and 1.36-fold higher in bioreactor. The combined treatment of squalene and chitosan resulted in the concentrations of total withanolides 2.13-fold and 1.66-fold higher in shake-flask and bioreactor cultures, respectively. The biomass of cell cultures showed reduction in growth up on elicitor treatment except aluminium chloride. Zhang et al. [39] reported that chitosan treated cell suspension cultures in *Taxus chinensis* showed maximum paclitoxel production whereas the cell growth declined due to chitosan inhibitor effect. Krzyzanowska et al. [40] stated that elicitor concentration and the time of incubation with elicitor are crucial for the elicitation process. Elicitor specificity, its concentration and time of its exposure, as well as the culture conditions and growth stage of the cultured cells influence the elicitation process [41]. Zhao et al. [42] proposed a hypothesis that cellular process and regulatory principles are involved in the activation of plant secondary metabolite biosynthesis. Accordingly, an extracellular or intracellular signal is perceived by a receptor on the surface of the plasma membrane or endomembrane; the elicitor signal perception initiates a signal transduction network that leads to activation or *de novo* biosynthesis of transcription factors, which regulate the expression of biosynthetic genes involved in plant secondary metabolism. The resulting enzymes catalyze the biosynthesis of target secondary metabolites. Many reports on polysaccharides and heavy metal ions elicitation in plant cell/organ cultures recorded enhanced secondary metabolites production with concurrent biomass reduction. For instance, supplementation of chitosan and cadmium chloride in the culture medium significantly enhanced tanshinone accumulation in *Salvia miltiorrhiza* cell suspension culture with a remarkable inhibition of cell growth [43]. Baldi and Dixit [38] stated that addition of chitosan resulted in reduction in cell growth and significant improvement in artemisinin content in cell suspension culture of *A. Annua* due to change in cell membrane permeability as evident from an increase in medium conductivity upon addition of chitosan. Mishra et al. [19] reported that cadmium stress altered enzymatic and non-enzymatic responses of ROS, sugar metabolism and contents of various biochemical constituents in the shoot culture of *W. somnifera* and suggested that these changes reflected their protective role during stress allowing cell growth to happen and tissue to live longer. Sabir et al. [12] suggested that withanolides are associated with membrane, acted as membrane-protectant under stress with less-prominent withanolide-related changes. In the present study, the elicitor treatment though reduced the growth of cells in culture enhanced withanolides contents.

In the secondary metabolites biosynthesis of plant cell cultures, precursor feeding at suitable concentrations with optimal exposure time can elevate the synthesis of secondary metabolites. But on the other hand, excess precursor concentration with improper exposure time may cause feedback inhibition to the metabolite pathway. Determination of appropriate precursor concentration in precursor-feeding is essential to achieve higher production of secondary metabolites [37]. Of various precursors tested, squalene at 6 mM increased the contents of withanolide A (1.37-fold), withanolide B (1.17-fold), withaferin A (1.35-fold), withanone (1.27-fold), 12-deoxy withanstramonolide (1.98-fold), withanosides IV (1.63-fold) and V (1.20-fold), whereas at higher concentrations (8–10 mM) its production was reduced when compared to control. Induction of withanolides by mevalonic acid was modest at 20% on a dry weight basis. The variations in uptake and degradation of squalene and mevalonic acid will manipulate the patterns and levels of accumulated steroidal lactones [44]. From the present investigation, the obtained results exhibited that squalene had a much better effect than mevalonic acid and cholesterol on withanolides synthesis. Chaurasiya et al. [45] suggested the significant contribution of DOXP pathway to withanogenesis in *W. somnifera*. Withanolides have been synthesized from MVA and/or DOXP pathway of isoprenogenesis through the intermediates, oxidosqualene, methylene iophenol, methylene cholesterol and compestrol which are also responsible for the synthesis of sterols and brassinosteroids [46]. The pathway indicated that squalene is metabolized from mevalonic acid. Hence, squalene can easily facilitate to participate in metabolic biosynthetic pathway of withanolides in large quantity than cholesterol. Our results indicate that when the concentration of each precursor in the culture medium was increased, the corresponding levels of all withanolides were enhanced during the exponential phase and then decreased during decline phase which ws either due to slow growth rate or cell lysis in suspension culture beyond 28 days. The reason by this indication that each precursor applied had both negative and positive impact on biosynthesis of withanolides in cell suspension culture of *W. somnifera*. There were numerous reports on precursor feeding strategies in plant cell/organ cultures to improve secondary metabolites production whereas the growth characteristics were considerably reduced upon precursor treatment depending upon the plant species tested. For example, inclusion of cholesterol and squalene in the culture medium confirmed extensive synthesis of digitoxin and digoxin in *Digitalis purpurea* culture, whereas their addition reduced the biomass accumulation [47]. Addition of mevalonic acid increased production of artemisinin in cell suspension culture of *A. annua* and its supplementation in the medium reduced the growth properties [38]. The present results exhibited that the optimization of precursor type, concentration and specific exposure time are indispensable to increase withanolides biosynthesis in cell suspension culture of *W. somnifera*.

Zhao et al. [48] postulated that the secondary metabolite production by combined treatments could enhance the stimulating effects each other by related mechanism(s). The present results suggested that the mechanism of elicitation and precursor feeding in the cell suspension culture promoted the major and minor withanolides synthesis. Each elicitor or precursor accelerated withanolides production by diverse ways, the mechanism for every treatment may be more intricate since it depends on the interactions between physiological effects caused by two treatments as observed by Zhao et al. [48] in *Catharanthus roseus* cell suspension culture. In the present study, squalene (6 mM) and chitosan (100 mg/l) influenced higher production of major and minor withanolides higher in both shake-flask and bioreactor cultures. The combined feeding of optimized concentrations of chitosan and squalene resulted in higher withanolides synthesis in both the culture systems than the individual feeding. Therefore, using this strategy with specific exposure time may be a practical way for higher withanolides production since this strategy could greatly improve withanolides production in a shorter culture period, and therefore reduce the process cost as suggested by Zhao et al. [48]. From the present study, it was confirmed that squalene interacted with chitosan or vice versa and promoted the biosynthesis of withanolides production. To our knowledge, this is the first report on the improved biosynthesis of all major and minor withanolides of *W. somnifera* in bioreactor by the influence of optimized culture conditions with combined addition of precursor and elicitor.

In conclusion, enhanced withanolides production was successfully established in cell suspension culture of *W. somnifera* up on combined treatment of squalene (6 mM) and chitosan (100 mg/l) [4 h and 48 h exposure times on 21^st^ day of culture, respectively] in plant cell culture medium with standardized levels of picloram (1 mg/l), KN (0.5 mg/l), L-glutamine (200 mg/l) and sucrose (5%) on 28^th^ day of culture in shake-flask culture and bioreactor. The present study revealed that withanolides contents varied considerably depending up on the type of elicitor or precursor in association with exposure time and culture age. This culture system established now will be useful in biochemical and bioprocess engineering for the feasible production of major and minor withanolides in large-scale using Industrial bioreactor.

## Supporting Information

Figure S1 **HPLC analyses of withanolides quantification in cell suspension culture of** ***W. somnifera*** **cultured in bioreactor.** (A) Standard withanolides (1–12 deoxy withanstramonolide; 2-withanferin A; 3-withanolide A; 4-withanone; 5- withanoside IV; 6-withanolide B;7- withanoside V). (B) Methanolic extract of cell suspension culture treated with chitosan and squalene in shake-flask culture. (C) Methanolic extract of cell suspension culture treated with chitosan and squalene in bioreactor culture.
(TIF)
