# Supplementary figures and images for: Enhanced Biosynthesis of Withanolides by Elicitation and Precursor Feeding in Cell Suspension Culture of Withania somnifera (L.) Dunal in Shake-Flask Culture and Bioreactor
Source: PLoS One. 2014 Aug 4;9(8):e104005. doi: 10.1371/journal.pone.0104005 (PMC4121209; doi:10.1371/journal.pone.0104005)

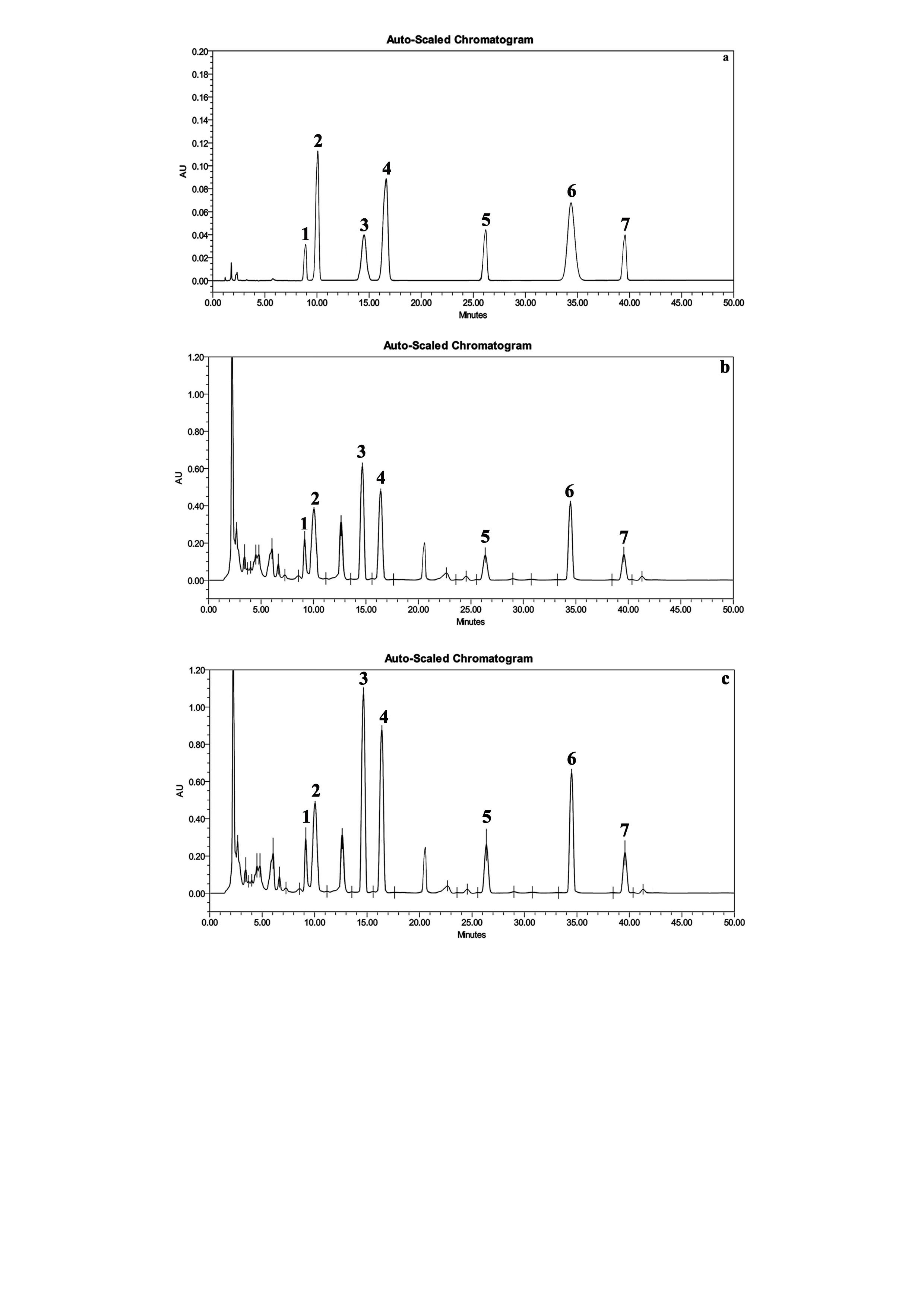

Supplement: Figure S1 — HPLC analyses of withanolides quantification in cell suspension culture of W. somnifera cultured in bioreactor. (A) Standard withanolides (1–12 deoxy withanstramonolide; 2-withanferin A; 3-withanolide A; 4-withanone; 5- withanoside IV; 6-withanolide B;7- withanoside V). (B) Methanolic extract of cell suspension culture treated with chitosan and squalene in shake-flask culture. (C) Methanolic extract of cell suspension culture treated with chitosan and squalene in bioreactor culture. (TIF) [file pone.0104005.s001.tif]
